# Evaluation of record linkage of two large administrative databases in a middle income country: stillbirths and notifications of dengue during pregnancy in Brazil

**DOI:** 10.1186/s12911-017-0506-5

**Published:** 2017-07-17

**Authors:** Enny S Paixão, Katie Harron, Kleydson Andrade, Maria Glória Teixeira, Rosemeire L. Fiaccone, Maria da Conceição N. Costa, Laura C. Rodrigues

**Affiliations:** 1 London School of Hygiene and Tropical Medicine, Keppel St, Bloomsbury, London, WC1E 7HT UK; 2 Instituto de Saúde Coletiva, Rua Basílio da Gama, s/n.Canela, Salvador, Bahia CEP 40110040 Brazil; 3 Departamento de Estatística, Av Ademar de Barros, s/n Ondina, Salvador, Bahia CEP 40170110 Brazil

**Keywords:** Data linkage, Routine data, Electronic health records, Linkage quality, Linkage accuracy, Stillbirth, Dengue

## Abstract

**Background:**

Due to the increasing availability of individual-level information across different electronic datasets, record linkage has become an efficient and important research tool. High quality linkage is essential for producing robust results. The objective of this study was to describe the process of preparing and linking national Brazilian datasets, and to compare the accuracy of different linkage methods for assessing the risk of stillbirth due to dengue in pregnancy.

**Methods:**

We linked mothers and stillbirths in two routinely collected datasets from Brazil for 2009–2010: for dengue in pregnancy, notifications of infectious diseases (SINAN); for stillbirths, mortality (SIM). Since there was no unique identifier, we used probabilistic linkage based on maternal name, age and municipality. We compared two probabilistic approaches, each with two thresholds: 1) a bespoke linkage algorithm; 2) a standard linkage software widely used in Brazil (*ReclinkIII*), and used manual review to identify further links. Sensitivity and positive predictive value (PPV) were estimated using a subset of gold-standard data created through manual review. We examined the characteristics of false-matches and missed-matches to identify any sources of bias.

**Results:**

From records of 678,999 dengue cases and 62,373 stillbirths, the gold-standard linkage identified 191 cases. The bespoke linkage algorithm with a conservative threshold produced 131 links, with sensitivity = 64.4% (68 missed-matches) and PPV = 92.5% (8 false-matches). Manual review of uncertain links identified an additional 37 links, increasing sensitivity to 83.7%. The bespoke algorithm with a relaxed threshold identified 132 true matches (sensitivity = 69.1%), but introduced 61 false-matches (PPV = 68.4%). *ReclinkIII* produced lower sensitivity and PPV than the bespoke linkage algorithm. Linkage error was not associated with any recorded study variables.

**Conclusion:**

Despite a lack of unique identifiers for linking mothers and stillbirths, we demonstrate a high standard of linkage of large routine databases from a middle income country. Probabilistic linkage and manual review were essential for accurately identifying cases for a case-control study, but this approach may not be feasible for larger databases or for linkage of more common outcomes.

## Background

Record linkage is the process used to bring together information recorded in different sources about the same individual or group of individuals [1]. Due to the growing availability of administrative population-based health databases, linkage has become an efficient and important research tool [2]. One research area for which linkage is particularly important is maternal and infant health, where linkage can facilitate increased understanding of how maternal health trajectories prior to and during pregnancy are associated with birth and later childhood outcomes [3]. Many countries use linkage of records from mothers and their babies to underpin both research and service evaluation [4–6].

High quality linkage is required so that robust results can be obtained from linked data. However, there are specific challenges in mother-baby linkage, particularly concerning data from low-middle income countries. Firstly, quality of linkage is limited by the availability of unique or well-completed identifiers, and by data quality (including truncated records and absent or ambiguous information [7, 8]. A further complication is that linkage of records belonging to different individuals is required (e.g. mothers and their babies), which limits the availability of common identifiers for linkage. Finally, when datasets do not overlap exactly, the expected number of matches is unknown, making it difficult to establish the expected number of records that should be linked [3].

There are two main approaches to linkage: deterministic and probabilistic. Deterministic linkage is a rule-based approach, typically using a unique identifier or set of common identifiers present in both files. Probabilistic linkage is useful when the quality of identifiers is not sufficient for a strict deterministic linkage due to missing values or typographical errors [9]. Probabilistic linkage combines evidence across a number of identifiers such as name, age and place of residence to calculate a match weight, representing the likelihood that two records belong to the same person, i.e., that they are a “true match” [10]. Match weights are used to classify records as links, non-links and uncertain links, generally by defining two thresholds [11]. The choice of threshold affects the number of “false-matches” (records from different individuals that are linked) and “missed-matches” (records from the same individual that fail to link) [12]. Probabilistic linkage has facilitated many studies in countries without a unique identifier [4–6].

The impact of linkage errors, in terms of bias in results of analysis, depends on the study in question [13]. For example, in many studies, it may be important to achieve a high match rate, so that the resulting linked data is representative of the source population and to avoid selection bias (especially if missed-matches are non-random, i.e. are more likely to occur in specific subgroups of records). In others, it may be more important to avoid false-matches. For example, in case-control studies, we may be more concerned with accurately establishing exposure status through linkage with a disease registry. This would require certainty that linked records really should have been linked, and unlinked records did not have the exposure.

In this study, we aimed to use linkage of national data sources to facilitate a case-control study of stillbirths in women who had notified symptomatic dengue during pregnancy. Linkage was required to establish exposure status in cases. In this scenario, it was important to: 1) prioritize true matches (i.e. high specificity of the exposure), whilst high sensitivity was less important; 2) retain sufficient cases for a reasonable sample size; and 3) verify absence of bias (i.e. understand whether any groups were more or less likely to be linked). This study presents an approach to preparing and linking mortality and morbidity data from routine data sources in Brazil, comparing the performance of different linkage methods for identifying exposure of pregnant women to dengue.

## Methods

### Datasets

We linked two routinely collected data sets: for dengue, notifications of infectious diseases (SINAN); for stillbirths, mortality (SIM), for 2009 and 2010.

1. **SINAN:** Notifiable Diseases Information System (Sistema de Informação de Agravos de Notificação/SINAN), containing individual-level data on all notified diseases.

#### Source

The Brazilian Ministry of Health has required notification of all cases of dengue seen in health facilities in Brazil. SINAN captures clinical cases of disease, through forms completed by any health professional who suspects dengue; this notification is compulsory in the country. After a dengue suspected case is identified, the Epidemiological Surveillance Service investigates cases in order to confirm or discard the suspicion based on laboratory results and the Brazilian definition of clinical epidemiological criteria of dengue: presence of fever and two or more of the following symptoms (retro-orbital or ocular pain, headache, rash, myalgia, arthralgia, leukopenia, or haemorrhagic manifestations) [14]. Forms include personal information on the patient (name, place of residence, age, marital status, and education) and on their disease (symptoms, laboratory tests and severity).

#### Completeness of linkage variables

Maternal name was 99.5% complete; municipality was 100% complete. Where age in years was missing (1.3% of records), we derived age from date of birth.

#### Data extraction

**Fig. 1:**
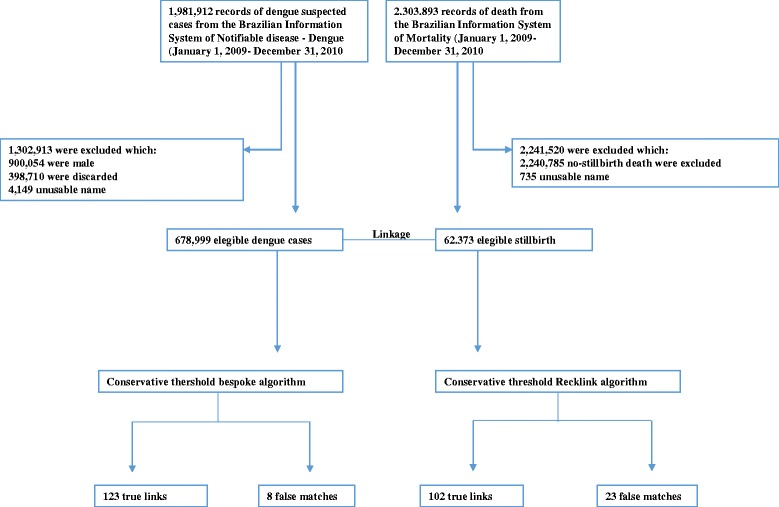
Number of records from Brazilian Information System of Notifiable Disease and Brazilian Information System of Mortality

Data were extracted from SINAN for all suspected dengue cases (*n* = 1,981,912 individuals). We excluded records for 900,054 men, and 398,710 cases discarded by the Epidemiological Surveillance services (Fig. 1).

2. **SIM:** Mortality Information System (Sistema de Informação sobre Mortalidade), containing individual-level data on all deaths including stillbirths.

#### Source

The Brazilian Ministry of Health requires notification of all patients who die, irrespective of place of death. Deaths are recorded using a death certificate, which is a legal document completed by physicians [15]. The definition of stillbirth as recorded in SIM is the death of a product of conception before the expulsion or complete extraction from the body of the pregnant woman, occurring from 22 weeks or weighing more than 500 g [15]. The form includes information on the mother (name, place of residence, age, marital status, education, whether she had a previous stillbirth or a child who died); and the pregnancy (length of gestation, type of delivery).

#### Completeness of linkage variables

Maternal name was complete in 98.8% of records; municipality was 100% complete. Age was complete in 84.6% of records.

#### Data extraction

Data on all deaths were extracted from SIM (*n* = 2,303,893 records). We kept only the stillbirths (*n* = 63,108).

### The process of linking

#### Data pre-processing

We excluded records without names, those with generic names such as “unknown” or “stillbirth”, records with only one name (e.g. “Maria”), and those name recorded as numbers: our final study population comprised 678,999 dengue notifications from SINAN and 62,373 stillbirths from SIM (Fig. 1). We searched automatically for improbable ages (e.g. 99 or age of the mother 1 year old) and set to null. We removed punctuation, deleted consecutive spaces, transformed known prefixes in the real names (e.g. Mr. ➔ Maria), deleted unknown prefixes, replaced names in upper case and dropped middle initials.

In Brazil, full names usually have several components, and we aimed to retain the discriminatory power of this variable. In our data, the mean number of names per mother was three (maximum eight). We derived distinct variables for first name, second name, and last name. Similarity between names recorded in SIM and SINAN was compared using the Jaro-Winkler string comparator [16]. The Jaro-Winkler string comparator counts the number of common characters between two strings and the number of transpositions of these common characters, producing similarity values varying between 1 (perfectly similar) and 0 [16]. We categorized the string comparator score as (0,0.85], (0.85,0.9], (0.9,0.95], (0.95,0.99] and (0.99,1].

#### Blocking

The total number of pairwise comparisons between SINAN and SIM would be prohibitively high: 184,922 × 31,867 = 5,892,909,374 in 2009 and 494,077 × 30,506 = 15,072,312,962 in 2010 (a dengue epidemic year). To decrease the number of pairwise comparisons, we only attempted to link women that resided in the same municipality (*n* = 5565 municipalities in Brazil in 2010). This process is called blocking; blocking by municipality assumes that records from different municipalities do not belong to the same women. We used this blocking scheme because we considered municipality to be very reliable, and unlikely to have changed between dengue in pregnancy and end of pregnancy.

#### Gold-standard data

We created a “Gold-standard” dataset using nine steps (Table 1). Firstly, we linked SIM and SINAN using deterministic linkage with exact agreement on full name and age. Subsequent steps relaxed the rules. Since mothers could be diagnosed during pregnancy in 1 year and give birth in the following year, and to allow for errors in recording, we allowed matches where there were differences of up to 2 years in age. To account for differences in the ways names were recorded, we separately looked for agreement on first and last name, first and second name, or second and last name. Each step was followed by manual review to exclude false-matches. Since some names were very common in the database (e.g. Maria), records with these names were only considered as a match where there was exact agreement on name and municipality, and age differed by no more than 1 year.

**Table 1:** Deterministic rules used to create the gold-standard database

Linkage rule	Number of links (%)
Full name and age^a^	46 (24.1%)
Age^a^ and combination of first and last name	13 (6.8%)
Age^a^ and combination of first and second name	9 (4.8%)
Age^a^ and combination of second and last name	19 (9.9%)
Full name	65 (34.0%)
First and last name	19 (9.9%)
First and second name	8 (4.2%)
Second and last name	12 (6.3%)
Age^a^ and Jaro-Winkler string comparator >0.95	0 (0%)
	**191**

^a^Age in years as recorded in the data or as derived from date of birth

#### Bespoke algorithm

For each record pair we calculated a probabilistic match weight based on two conditional probabilities: the probability of agreement given records belong to the same mother-baby pair (m-probability; P (agreement|match)) and the probability of agreement given records belong to different mother-baby pairs (u-probability, P (agreement|non-match)).

M-probabilities for each identifier were estimated from the true matches in the gold-standard dataset. U-probabilities were calculated based on a list of non-matches, created from all pairwise comparisons of records within SINAN, excluding those belonging to the same individual. We used the SIINAN database to calculate the u-probabilities, as it was the larger of the two databases.

Frequency-based weights were calculated for each category of Jaro-Winkler score comparator [16] (for name of the mother) and year of age. Weights were also separately calculated for the five most frequently occurring names in the data (Maria, Ana, Santos, Souza and Oliveira).

Since we were linking different subjects (the mother and her stillbirth), and because the exposure (dengue during pregnancy) could have happened up to 9 months before the outcome (stillbirth) there were timing issues to consider. Two records could differ in time by 9 months and could bridge over the calendar year; some mothers would have the birthday between the data of dengue and the date of the stillbirth. To allow for this, we estimated different weights according to the similarity of age across datasets: equal ages, age differing by 1 year, age differing by 2 years, and ages differing by more than 2 years (Table 2).

**Table 2:** Comparison of linkage strategies for the bespoke algorithm and *ReclinkIII*

	Bespoke algorithm	*ReclinkIII*
Manipulation of names	• Multiple variables created for first name, second name, and last name	• Variables created for first and last name
Blocking	• Municipality	• Soundex for name + municipality
Calculation of *m* and *u* probabilities	• *m*-probability: calculated using true-matches in gold-standard • *u-* probability: calculated using non-matches in SINAN	• *m*-probabilities = 0.9 • *u*-probabilities = 0.1
Match weight calculation	• Separate weights calculated for the five most common names • Agreement on name classified using Jaro-Winkler string comparator • Different weights calculated according to closeness of age.	• Did not account for common names • Levenshtein string comparator • Did not account for timing issues

Match weights were calculated by summing the ratio of m-probabilities and u-probabilities across different identifiers [1]. The algorithm was implemented in Stata and R.

#### ReclinkIII algorithm

As we were using Brazilian data, we compared the accuracy of our bespoke linkage algorithm with a widely used software for linking data in Brazil, called *Reclink* version III.

*ReclinkIII* calculates match weights in 3 steps: 1) Manipulation of names; 2) Blocking (*Reclink* matches pairs within blocks of similar names defined by soundex; in addition we programmed *ReclinkIII* to block by municipality); 3) Match weight calculation (the final score is the sum of the weighted scores of each field, e.g. name and age).

*ReclinkIII* applies the Levenshtein string comparator to compare names [17]. The Levenshtein string comparator is defined as the minimum number of insertions, deletions, or substitutions necessary to change one string into the other, the values varying between 1 (perfect similarly) and 0 (total disagreement). The m-probabilities and u-probabilities were based on default values as suggested by the software: m-probabilities = 0.9 and u-probabilities = 0.1 for all identifiers (Table 2) [18].

#### Classification of links

Records pairs were ordered by match weight and manually inspected to identify threshold values to classify comparison pairs as non-links, links and uncertain links. The number of expected matches was unknown, because we did not know a priori how many of the mothers in SIM should link to a stillbirth in SINAN. Therefore, we explored two different threshold choices for each algorithm. We first chose a conservative threshold, aiming to exclude as many as false-matches as possible (high positive predictive value). We then chose a relaxed threshold, aiming to capture as many of the true matches as possible (high sensitivity). Any records above the cut-off threshold were classified as links. For the best performing approach, we manually inspected uncertain links to determine whether or not they belonged to the same mother-baby pair.

### Statistical analysis

For both the bespoke and the *ReclinkIII* algorithm, and for each threshold (conservative and relaxed), we estimated the sensitivity and positive predictive value (PPV) by comparing linkage results with the gold-standard dataset. Since we expected the number of links to be very small in comparison to the size of the datasets, we did not calculate specificity or negative predictive value (as these measures would be consistently high). To account for in-sample optimism, we also present average estimates based on ‘leave one out’ cross classification.

For the best performing algorithm, we examined which characteristics were associated with false-matches and missed matches. Categorical variables were compared between groups with Chi^2^ test or Fisher’s exact test. A two-sided *P* value of less than 0.05 was considered to indicate statistical significance. We examined maternal age (<20, 20–35, >35 years), maternal education (illiterate, 1–3 years, 4–7 years, 8–11 years and more than 11 years), previous stillbirths or abortions (yes/no), gestational age (less than 22 weeks, 22–27 weeks, 28–31 weeks, 32–36 weeks, 37–41 weeks, more than 42 weeks) and weight when the stillbirth occurred (> = 2500, 1500–2500, <1500 g). Stata version 14.1 was used for the statistical analyses.

## Results

### Gold-standard

Of the 678,999 eligible dengue cases in SINAN for 2009–2010, 191 were linked to a stillbirth record using the nine-step gold-standard algorithm (Table 1).

### Bespoke algorithm

The conservative threshold was set to a match weight of 21, and resulted in 131 links (Fig. 1). Comparison with the gold-standard identified 8 false matches and 68 missed-matches, giving a sensitivity of 123/191 = 64.4% and a PPV of 123/131 = 93.9% (Table 3). Adjusting for in-sample optimism gave values of 64.4% and 94.1% respectively.

**Table 3:** Performance of linkage algorithms and thresholds

	Bespoke algorithm		*ReclinkIII* algorithm	
	Conservative threshold = 21	Relaxed threshold = 20	Conservative threshold = 12	Relaxed threshold = 10
N linked	131	193	125	788
N true links	123	132	102	114
N false-matches	8	61	23	674
N missed-matches	68	59	89	77
Sensitivity % (95% CI)	64.4 (57.2–71.2)	69.1 (62.0–75.6)	53.4 (46.1–60.6)	59.7 (52.4–66.7)
Positive predictive value % (95% CI)	93.9 (86.6–96.3)	68.4 (61.3–74.8)	81.6 (73.7–87.9)	14.5 (12.1–17.1)

The relaxed threshold was set at 20 and resulted in 193 links. Comparison with the gold-standard identified 132 true links, giving a sensitivity of 132/191 = 69.1%, and 61 false-matches, giving a PPV of 132/193 = 68.4% (Table 3). Adjusting for in-sample optimism gave values of 69.1% and 68.4% respectively.

### ReclinkIII

The conservative threshold was set at 12 and resulted in 125 links (Fig. 1). Comparison with the gold-standard identified 102 true links, giving a sensitivity of 102/191 = 53.4%, and 23 false-matches, giving a PPV of 102/125 = 81.6% (Table 3). Adjusting for in-sample optimism gave values of 53.4% and 81.8% respectively.

The relaxed threshold was set at 10 and resulted in 788 links. Comparison with the gold-standard identified 114 true links, giving a sensitivity of 114/191 = 59.7%, and 674 false-matches, giving a PPV of 114/788 = 14.5% (Table 3). Adjusting for in-sample optimism gave values of 59.7% and 14.4% respectively.

### Linkage errors

Missed-matches and false-matches had a higher proportion of missing data (Table 4). Linkage errors were not associated with any recorded study variables, although there was a suggestion that mothers aged <20 were slightly less likely to link (*p* = 0.047) (Table 4).

**Table 4:** Associations between linkage accuracy (using the bespoke algorithm) and characteristics of the cohort

	True matches *N* = 123 n (%)	Missed-matches *N* = 68 n (%)	OR (95% CI)	*p*-value	False-matches *N* = 8 n (%)	OR (95% CI)	*p*-value
Age of the mother in years							
< 20	25 (20.3)	19 (27.9)	1	*p* = 0.047	-		*p* = 0.095
20–35	67 (54.5)	30 (44.1)	1.7 (0.8–3.5)		8 (100)		
> 35	22 (17.9)	7 (10.3)	2.3 (0.8–6.7)		-		
Missing	9 (7.3)	12 (17.5)			-		
Maternal literacy							
Illiterate	7 (5.7)	5 (7.3)	1	*p* = 0.113	-	1	*p* = 0.617
1–3 years	8 (6.5)	4 (5.9)	1.4 (0.3–7.5)		1 (12.5)	0.9 (0.4–1.9)^a^	
4–7 years	32 (26.0)	14 (20.6)	1.6 (0.4–6.0)		2 (25.0)		
> 8 years	34 (27.6)	20 (29.4)	1.2 (0.3–4.3)		4 (50.0)		
> 11 years	19 (15.4)	3 (4.4)	4.5 (0.8–24.1)		-		
Missing	23 (18.7)	22 (32.3)			1 (12.5)		
Previous fetal death or abortion							
No	36 (29.3)	17 (25.0)	1	*p* = 0.574	2 (25.0)	1	*p* = 0.966
Yes	59 (48.0)	31 (45.6)	0.9 (0.4–1.8)		4 (50.0)	1.2 (0.2–7)^a^	
Missing	28 (22.7)	20 (29.4)			2 (25.0)		
Gestational age							
< 22 weeks	9 (7.3)	2 (2.9)	1	*p* = 0.248	-		*p* = 0.492
22–27 weeks	29 (23.6)	18 (26.5)	0.3 (0.1–1.8)		3 (37.5)	1	
28–31 weeks	21 (17.8)	11 (16.2)	0.4 (0.1–2.3)		3 (37.5)	0.7 (0.4–1.3)^a^	
32–36 weeks	26 (21.1)	19 (27.9)	0.3 (0.1–1.5)		2 (25.0)		
37–41 weeks	28 (22.8)	9 (13.2)	0.7 (0.1–3.8)		-		
≥ 42 weeks	2 (1.6)	-			-		
Missing	8 (6.5)	9 (13.2)			-		
Birth or death weight							
≥ 2500	23 (18.7)	16 (23.5)	1	*p* = 0.798	-		*p* = 0.165
1500–2500	26 (21.2)	16 (23.5)	1.1 (0.4–2.7)		-		
< 1500	64 (52.0)	31 (45.6)	1.4 (0.6–3.0)		7 (87.5)		
Missing	10 (8.1)	7 (7.3)			1 (12.5)		

^a^Due to the small number of observations, we used only two categories, the first category without missing value as the reference one

### Manual review

For the bespoke linkage algorithm with the conservative threshold, uncertain links were defined as those with weights between 16 and 21. Records with these weights were classified through manually inspecting each record to determine whether or not they belonged to the same person. This added 37 links, resulting in 160 true links, increasing the sensitivity to 83.7% and bringing the total number of linked records to 168.

## Discussion

Our study demonstrates that high-quality linkage of records belonging to different individuals in large routine databases from a middle-income country can be achieved, without unique identifiers. Importantly, for the purposes of establishing exposure to dengue for a case-control study, we were able to accurately identify links with a low false-match rate by using a bespoke linkage algorithm designed to overcome the challenges of linking different individuals in national data from Brazil. Although the restricted number of common variables for mothers and stillbirths limited the number of links that could be automatically detected, manual inspection allowed us to greatly improve sensitivity; however, this approach is resource-intensive and may not be feasible for larger databases, such as live births in Brazil with 3 million records a year. Our comparison of missed-matches and true-matches indicate that linkage errors occur randomly, and are unlikely to introduce bias into our analyses.

We show that linkage between large administrative datasets is complex and requires a number of steps. Our description of these steps (and commands listed in the annex) are available as guidance to others aiming to link similar data sources. Although we did not have a readily available training dataset (where the true match status of each record pair was known), we were able to create a gold-standard dataset from which to derive an appropriate match weight algorithm, and to evaluate the accuracy of both linkage methodologies. This was possible in our study because we had access to identifiable data, and could examine records manually, but is not always the case, when data from clinical and identifiers information are separated to protect the patient privacy [19].

The bespoke algorithm created specifically to link this dataset achieved higher linkage quality that the off-the-shelf program *RecLinkIII,* which has been widely used to perform linkage in Brazil and which has previously been shown to have high sensitivity and specificity [7, 20–22]. *RecLinkIII* appears to work less well when there are a limited number of variables available to perform the linkage, as demonstrated in our study and a similar study by Coutinho et al. [23], which obtained a sensitivity of 60.9% and 72.8% (without and including the uncertain area respectively). The *RecLinkIII* program uses the Levenshtein string comparator, which according with Freire [24] is not the most effective option to compare names in Brazil. The Jaro-Winkler string comparator, used within our bespoke algorithm, has been shown to give the best results when compared with other string comparators to link names in Brazil [24]. Other string comparators may be more appropriate in other situations [25]. The improved performance of the bespoke linkage algorithm was also likely due to our derivation of frequency-based match weights and the fact that we allowed weights to differ for common names and differences in age. Additional strengths of our approach were that the data were rigorously prepared to ensure that information belonging to the same mothers and babies could be linked; we performed the validation study in a large sample size that allowed evaluation of two thresholds and comparison with a commercially available software, and because we had additional information about the characteristics of the mothers included in the study we were able to check for bias.

An important aspect of linkage quality is the choice of threshold. The investigator, keeping in mind that different thresholds are more appropriate for different study questions, must make this choice. In our study, where linkage aimed to provide information on exposure (dengue during pregnancy) [26] we chose a conservative threshold to minimise false-matches. We showed that more relaxed thresholds added a number of false-matches, drastically decreasing the positive predictive value, without substantially increasing sensitivity. This is because stillbirth is a rare outcome, and there was a large number of real non-matches.

This study has a number of limitations. Although a gold-standard dataset was used to measure the linkage accuracy, there remains scope for linkage errors to occur: we may not have identified all missed-matches due to missing data on some records. Our estimates of sensitivity should therefore be interpreted with caution, and should not be assumed to apply to other datasets. Due to the low sensitivity of our linkage approach, our study should not be used to estimate stillbirth rates for women exposed to dengue during pregnancy. Our analysis of the association between study characteristics and linkage error may have been limited due to low power. A further consideration for case-control studies is whether linkage of cases and controls (to the exposure) has similar accuracy. We did not address the accuracy of linkage to live births in this study, but will evaluate this in future research.

## Conclusion

To make best use of linked data, it is important to evaluate the quality of linkage processes and to understand the limitations and bias that errors in linkage could introduce in the research results [27, 28]. Validation studies are therefore useful for assessing whether probabilistic matching of such records is effective and whether results are reliable. We present this validation study to add to the limited existing evaluations of data linkage from middle income countries using a limited number of identifiers. We show that it is possible to achieve a high standard of linkage between different individuals within administrative data from Brazil, specifically for the purposes of accurately stillbirths exposed to dengue [26]. Our results highlight that bespoke linkage algorithms perform better than off-the-shelf software, and that manual review can be a valuable tool for improving sensitivity.

## Abbreviations

PPV: Positive predictive value
*ReclinkIII*: Software to link data used in Brazil
SIM: Mortality information system
SINAN: Notifiable diseases information system
